# Diminished Metal Accumulation in Riverine Fishes Exposed to Acid Mine Drainage over Five Decades

**DOI:** 10.1371/journal.pone.0091371

**Published:** 2014-03-24

**Authors:** Ross A. Jeffree, Scott J. Markich, John R. Twining

**Affiliations:** 1 School of the Environment (C3), University of Technology Sydney, Sydney, NSW, Australia; 2 Aquatic Solutions International, Collaroy, NSW, Australia; 3 Austral Radioecology, Oyster Bay, NSW, Australia; CINVESTAV-IPN, Mexico

## Abstract

Bony bream (*Nematalosa erebi*) and black catfish (*Neosilurus ater*) were sampled from the fresh surface waters of the Finniss River in tropical northern Australia, along a metal pollution gradient draining the Rum Jungle copper/uranium mine, a contaminant source for over five decades. Paradoxically, populations of both fish species exposed to the highest concentrations of mine-related metals (cobalt, copper, lead, manganese, nickel, uranium and zinc) in surface water and sediment had the lowest tissue (bone, liver and muscle) concentrations of these metals. The degree of reduction in tissue concentrations of exposed populations was also specific to each metal and inversely related to its degree of environmental increase above background. Several explanations for diminished metal bioaccumulation in fishes from the contaminated region were evaluated. Geochemical speciation modeling of metal bioavailability in surface water showed no differences between the contaminated region and the control sites. Also, the macro-nutrient (calcium, magnesium and sodium) water concentrations, that may competitively inhibit metal uptake, were not elevated with trace metal contamination. Reduced exposure to contaminants due to avoidance behavior was unlikely due to the absence of refugial water bodies with the requisite metal concentrations lower than the control sites and very reduced connectivity at time of sampling. The most plausible interpretation of these results is that populations of both fish species have modified kinetics within their metal bioaccumulation physiology, via adaptation or tolerance responses, to reduce their body burdens of metals. This hypothesis is consistent with (i) reduced tissue concentrations of calcium, magnesium and sodium (macro-nutrients), in exposed populations of both species, (ii) experimental findings for other fish species from the Finniss River and other contaminated regions, and (iii) the number of generations exposed to likely selection pressure over 50 years.

## Introduction

Over the next 50 years, agricultural and industrial growth in major river basins, coupled with greater population densities in coastal watersheds, will increase environmental contamination [1] and threaten biodiversity [2]. The adaptive responses of fisheries to long-term contaminant exposure have implications for resulting edible tissue concentrations and the health and welfare of those dependent on fish for subsistence, livelihood or consumption as ‘bush tucker’, ie. native foods used by Aboriginal communities. Aquatic systems with well-established pollution histories over multiple decades can uniquely function as natural laboratories, to give insights into these likely environmental futures.

**Figure 1:**
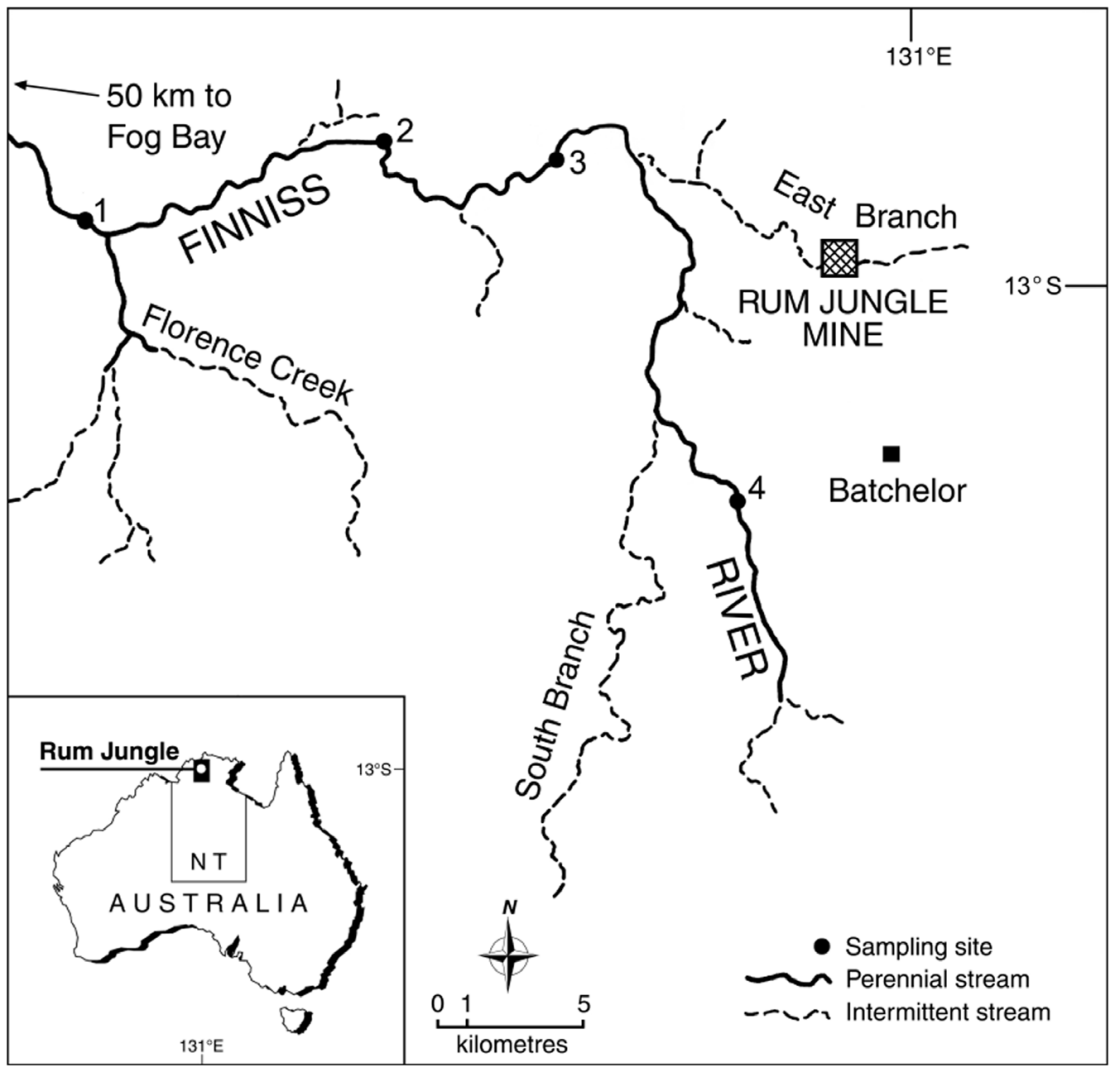
Location map. Location map showing the four sampling sites in the Finniss River. Sites 1 and 4 are uncontaminated (control) sites. Site 2 represents the pre-remediation extent of contaminant impact in the main river and site 3 is the most contaminated site.

The Finniss River in northern Australia (Figure 1) is a good example of a natural laboratory. It has been continually exposed to acid rock drainage (ARD) from the Rum Jungle mine site since the 1950s. Exposure to metal contaminants at low pH caused severe detriment to fish diversity and abundance, including regular fish-kills [3]. The system has been physico-chemically characterized to a level that rarely occurs (c.f. Restronguet Creek (UK), Foundry Cove (USA) or Sudbury Lakes (Canada)) and which consequently enhances the validity of biological assessments. Specifically, this site has had a well-defined history, over decadal periods, comprising quantified annual loads of the major contaminants and other water quality parameters, mainly based on daily water samples, as well as longitudinal profiles of contaminants in water and sediment. The corresponding extent and severity of biotic impact has also been determined, particularly for fish diversity, abundance and community structure [3], [4], [5], [6], [7], [8].

Following remedial work at the Rum Jungle mine site in the 1980s, fish communities in the Finniss River recovered such that multivariate statistical analysis detected no significant (*P*>0.05) differences in community structure between variably contaminated and uncontaminated sites, even though considerable contamination was still being delivered to the river [6], [8]. Some ecological impacts have remained. For example, benthic macroinvertebrate abundances in contaminated regions of the river system are relatively reduced when contaminants are delivered from the mine site during the wet season (November to March) [7], but also show recovery to values equivalent to uncontaminated regions during the dry season (after the cessation of annual contaminant input); thus providing a food source for fishes and exposure pathway for metals.

The post-remedial contaminant status of the recovered fish fauna is of increasing significance to the traditional Aboriginal owners. Their environmental values are now receiving much greater consideration in setting new remedial targets for the Rum Jungle mine site and its receiving waters [9]. The present study determined key metal (cobalt (Co), copper (Cu), manganese (Mn), lead, (Pb), nickel (Ni), uranium (U) and zinc (Zn)) levels in bone, liver and muscle (flesh) of two larger fish species, bony bream (*Nematalosa erebi*: Family Clupeidae) and black catfish (*Neosilurus ater*: Family Plotosidae), in the Finniss River. These fish are traditional Aboriginal “bush tucker” items and have been abundant in the contaminated region of the river since mine site remediation, despite previously having the least abundant populations [6]. The relationship between metal concentrations in tissues of both fish species and those in surface waters and sediments was also investigated.

## Materials and Methods

### Ethics statement

The sampling of fish from the Finniss River was authorized by the Northern Territory Department of Land Resource Management using methods approved by the Australian Nuclear Science and Technology Organisation Animal Care and Ethics Committee - which are consistent with the latest guidelines for ethical field sampling of fish [10]. The two selected fish species were neither protected nor endangered. No specific permissions were required for the sampling of sediment or water from each study site.

### Study site

The Finniss River is approximately 140 km long, is divided into a long fluvial and a small (3 km) tidal section, and receives inflow from several small tributaries (Figure 1). Flow in the Finniss River reflects a monsoonal climate with summer rainfall (1500 mm average annual rainfall between November and March) and winter drought. This type of rainfall pattern means that flow in tributaries is ephemeral, with surface flow ceasing soon after the end of the wet season, leaving a series of permanent waterbodies (known as billabongs) along the Finniss River.

The Rum Jungle Cu/U mine, Australia’s first fully operational U mine (1952–1971), is located on the East Branch of the Finniss River (Figure 1). At the time of its development, there was no legislative requirement for mining projects to undergo environmental impact assessment. Acid rock drainage, resulting from the oxidation of sulfide minerals in the waste rock, led to severe biological degradation of freshwater habitats downstream of the mine site [4]. This ecological impact was due to elevated concentrations of Co, Cu, Mn, Ni, Pb, U, Zn and sulfate in low pH (2–4) water. Rehabilitation of the mine between 1982 and 1985 resulted in a substantial (40–70%) reduction in the measured annual loads of Cu, Zn, Mn and sulfate exported by the East Branch of the Finniss River, that drains the mine site, as well as an increase in stream pH to 6.5 [6].

Four study sites were selected along the Finniss River (S 13.019, E 130.962; Figure 1), representing *a priori* contaminated and uncontaminated regions. The uncontaminated regions of the river were represented by site 1 (30 km downstream of the East Branch confluence, but also downstream of the perennial spring-fed Florence Creek tributary that dilutes and flushes downstream any contaminants present), and site 4 (15 km upstream of the East Branch confluence). The most metal-contaminated site (site 3, 4–5 km downstream from the East Branch) suffered repeated fish kills before mine site remediation [4]. The other metal-contaminated site (site 2, 15 km downstream of the East Branch confluence) had contaminant concentrations that significantly reduced fish diversity and abundance before mine site remediation [4], [6], but contaminant concentrations were, and remained, lower than at site 3.

### Fish sampling and preparation

Mature adult bony bream (*N. erebi*) and black catfish (*N. ater*) of similar sizes and ages (Table S1) were sampled at each site using a standardized set of gill nets (50 mm mesh size) in October 2003. Gender (male or female) and reproductive condition were determined from a visual inspection of the gonads, (based on dissection). Muscle, liver and bone (rib) tissues were dissected from each fish, thoroughly rinsed with deionised (Milli-Q, 18 MΩ cm^−1^) water and oven-dried at 40°C to a constant measured weight. A pre-weighed, homogenised, sub-sample of each tissue was then solubilised in concentrated nitric acid (7 mL) and hydrogen peroxide (2 mL) using a microwave digestion system (Milestone ETHOS 1). The resulting clear digest solution was cooled and made-up to 50 mL with deionised water.

### Sediment sampling and preparation

A composite sample of oxic surface sediment (0–2 cm depth) was randomly collected from several square meters of stream bottom at each site using a polypropylene scoop and stored in an acid-cleaned polyethylene container at 4°C. Homogenised whole sediments were wet-sieved through a 2 mm nylon mesh before being oven-dried (40°C) to a constant measured weight. Sub-samples (0.5 g) were extracted with 1 M HCl (20 mL) for 4 h (where a steady-state concentration was reached for all metals) at room temperature (22°C) in polypropylene vials using orbital agitation, filtered (Whatman GF/F) and made-up to 50 mL with deionised water.

### Surface water sampling and preparation

Surface water (at a depth of 40−60 cm) was sampled at each site with an acid-cleaned low density polyethylene bottle (1 L) using a two-person “clean” handling protocol [11]. Sub-samples were filtered using a 0.4 μm pre-cleaned polycarbonate filter (Nuclepore) and acidified (pH<2) using concentrated nitric acid (BDH).

### Chemical analyses

The pH and conductivity of surface waters (sites 1–4) was measured using a YSI model 63 hand held instrument. The concentrations of Al, Cd, Co, Cu, Mn, Ni, Pb, U and Zn in the fish tissue and sediment digests and/or surface waters were measured using inductively coupled plasma mass spectrometry (ICPMS, HP Agilent 4500), whereas Ca, Mg, Na, K, Fe and Si were measured using inductively coupled plasma atomic emission spectrometry (Varian Vista AX). A multi-metal calibration standard and a reagent blank were analysed with every ten samples to monitor signal drift. For all metals, the signals changed by less than 8%, but typically 3–5%. Where ICPMS was used, gallium, indium and rhenium were employed as internal standards to correct for any non-spectral interferences.

Chloride (Cl), sulfate (SO_4_), nitrate (NO_3_) and phosphate (PO_4_) were measured using ion chromatography (Dionex DX600). Hardness was calculated using standard method 2340B (Hardness (mg CaCO_3_/L)  = 2.47[Ca] + 4.11[Mg], where Ca and Mg are expressed in mg/L; [12]). Alkalinity was measured using potentiometric titration, following standard method 2320B for low alkalinity waters [12]. Bicarbonate concentrations were determined nomographically from alkalinity measurements using standard method 4500-CO_2_ [12]. Dissolved organic carbon was measured by ultraviolet-persulfate oxidation (Tekmar Dohrmann Pheonix 8000 TOC analyser) following standard method 5310C [12]. Suspended particulate matter determined gravimetrically following standard method 2540D [12].

Standard reference materials (SRM: National Research Council of Canada dogfish muscle (DORM-2) and liver (DOLT-3), stream sediment (STSD 1 and STSD 4), and riverine water for trace metals (SLRS-4)), sample replicates, and field blanks/reagent blanks were used to evaluate analytical accuracy, precision and limits, respectively. The mean concentrations of metals in the SRMs were within their certified ranges. For replicate samples and SRM, the percentage coefficient of variation was <10% for all metals.

### Geochemical speciation modeling

The speciation of Co, Cu, Mn, Ni, U and Zn in the surface waters of the Finniss River (sites 1–4) was calculated using both WHAM (version 7.0; [13]) and Visual MINTEQ (version 3.0; [14]). The inorganic equilibrium constants used in both models were derived from [15]. Metal binding with dissolved organic matter (DOM) was calculated using three independent sub-models (Humic Ion-Binding Model VII – incorporated into WHAM, the Stockholm Humic Model and the NICA Donnan Model – both incorporated into Visual MINTEQ). It was assumed that the ratio of active DOM/DOC was 1.4 and that 100% of the active DOM was fulvic acid – this is based on DOM containing 50% carbon (DOC) by mass with an active 70% fulvic acid concentration (a mean value based on studies by [16], [17], [18], [19], [20]). The generic acid-base properties and proton binding constants of fulvic acid used in the NICA Donnan Model were revised according to [21]. The speciation models also account for any minerals that may precipitate or any metal binding/adsorption to iron and/or aluminium (oxy)hydroxides, if applicable. The input parameters for both speciation models were based on measured water chemistry data (Table 1).

**Table 1:** Surface water chemistry of the Finniss River^a^.

	Site 4 (–15 km)	Site 3 (4 km)	Site 2 (15 km)	Site 1 (30 km)	WQG^b^
pH	7.3 (0.3)	7.2 (0.3)	7.5 (0.3)	5.5 (0.1)	—
Conductivity (μS/cm)	222 (36)	241 (40)	239 (41)	19 (1.8)	—
SPM^c^ (mg/L)	1.2 (0.4)	1.0 (0.3)	1.1 (0.4)	1.0 (0.3)	—
Hardness (mg/L as CaCO_3_)	90 (33)	95 (34)	98 (36)	4.3 (0.5)	—
Na (mg/L)	2.4 (0.4)	3.2 (0.4)	2.9 (0.4)	1.8 (0.2)	—
K (mg/L)	0.70 (0.12)	1.0 (0.2)	1.0 (0.2)	0.52 (0.05)	—
Ca (mg/L)	12 (4.7)	12 (4.6)	13 (4.8)	0.51 (0.06)	—
Mg (mg/L)	15 (5.9)	16 (6.3)	16 (6.4)	0.73 (0.09)	—
Fe (μg/L)	225 (35)	320 (40)	285 (36)	190 (28)	—
Al (μg/L)	45 (5.9)	63 (8.5)	54 (6.3)	30 (3.8)	—
Mn (μg/L)	27 (5.1)	156 (29)	63 (10)	28 (4.5)	1700
Cu (μg/L)	0.80 (0.16)	**35** (6.3)	**4.9** (1.2)	0.67 (0.15)	3.7^d^
Zn (μg/L)	0.75 (0.17)	**42** (7.5)	6.6 (1.6)	0.64 (0.15)	21^d^
Ni (μg/L)	0.54 (0.16)	17 (2.9)	3.1 (0.65)	0.56 (0.14)	29^d^
Co (μg/L)	0.50 (0.14)	16 (2.7)	2.7 (0.59)	0.47 (0.11)	—
U (μg/L)	0.14 (0.03)	1.1 (0.2)	0.35 (0.06)	0.11 (0.03)	5.0
Pb (μg/L)	0.083 (0.01)	0.15 (0.02)	0.10 (0.02)	0.078 (0.01)	15^d^
Cd (μg/L)	0.045 (0.02)	0.058 (0.02)	0.048 (0.02)	0.042 (0.02)	0.56^d^
HCO_3_ (mg/L)	103 (35)	108 (36)	110 (38)	4.1 (0.5)	—
Si(OH)_4_ (mg/L)	15 (1.9)	13 (1.5)	14 (1.4)	19 (2.0)	—
Cl (mg/L)	6.2 (1.2)	7.8 (1.5)	7.2 (1.4)	4.0 (0.6)	—
SO_4_ (mg/L)	2.6 (0.3)	4.5 (0.6)	3.1 (0.4)	0.28 (0.03)	—
NO_3_ (μg/L)	11 (1.7)	15 (2.1)	13 (1.7)	10 (1.1)	—
PO_4_ (μg/L)	5.2 (0.8)	5.6 (1.0)	4.9 (0.8)	3.4 (0.4)	—
DOC^e^ (mg/L)	3.4 (0.5)	3.1 (0.6)	3.5 (0.6)	2.3 (0.3)	—

a Mean (and standard deviation) values for surface waters of the Finniss River from 1986 to 2005 (n = 8−78). Data were obtained from this study, as well as published and unpublished reports by government authorities and private consultants. Values represent filtered (0.4 μm) concentrations averaged over the wet and dry season.

b. Water quality guideline (WQG) values for slightly-moderately disturbed aquatic ecosystems [24]. Metal concentrations that exceed the WQG values are indicated with bold text.

c Suspended particulate matter.

d Corrected for a water hardness of 95 mg/L as CaCO_3_ using the algorithm given in [24].

e Dissolved organic carbon.

### Annual cycle of surface water contamination

Daily measurements of flow rates and contaminant water concentrations that were used to determine annual contaminant loads delivered to the Finniss River, post-remediation, were also employed to estimate the typical annual cycle of contaminant water levels at the most contaminated site (site 3). Analysis of data for the 1994*−*1995 wet season (representative of wet season data from 1989–2004) focused on Cu, considered to be the most toxic metal, and was used to estimate its peak and average surface water concentration during the period of contaminant inflow to the Finniss River from the East Branch.

### Data analyses

One-way analysis of variance (ANOVA) was used to compare the concentrations of each trace metal (Co, Cu, Mn, Ni, Pb, U and Zn) and macro-nutrient (Ca, Mg and Na) for each selected tissue (bone, liver and muscle) and fish species (bony bream and black catfish) amongst the four sample sites. Where ANOVA indicated significant differences, pairwise comparisons of mean tissue metal concentrations (e.g. site 1 vs site 3, etc) were performed using the Tukey Honest Significant Difference test [22]. Linear regression analysis was used to investigate the relationship between the concentrations of Co, Cu, Mn, Ni, Pb, U and Zn in bone, liver or muscle of bony bream or black catfish, and (i) the filtered concentrations of Co, Cu, Mn, Ni, Pb, U and Zn in surface waters, or (ii) the concentrations of Co, Cu, Mn, Ni, Pb, U and Zn in surface sediments. The assumptions of ANOVA and linear regression were tested [22], and model adequacy was confirmed in all cases using either raw or transformed (log_10_) data. Significance was tested at the *P* = 0.05 level.

The similarities among individual fish based on their multi-metal concentrations in muscle, liver and bone were determined using multivariate analyses (PRIMER v6: [23]). The objectives were to determine (a) whether individuals of each fish species reflected the physico-chemistry at the sites of capture, based on their tissue metal concentrations, and (b) the relative differences in tissue metal concentrations between fishes from the most contaminated site (3) or the less contaminated site (2) and the uncontaminated sites (1 and 4). Data were log (n+1) transformed and normalized (to account for the large differences in tissue concentrations amongst metals) prior to non-metric multidimensional scaling (nMDS) analysis, where Euclidean distance was used as the measure of dissimilarity. Two-way crossed analysis of similarities (ANOSIM) was also performed on each of the six resemblance matrices to test for exposure and geographical location effects amongst sampling sites.

## Results

### Fish metrics

Total lengths and ages of bony bream and black catfish were not significantly (*P*>0.05) different amongst sites (Table S1). The reproductive condition of all individuals for both fish species was classified as maturing, where the testes of males were swollen, elongated and grey/white in appearance, and the ovaries of females were swollen and rounded with developing yellow eggs visible.

### Metal concentrations in surface waters and sediments

The surface water chemistry at each site is provided in Table 1. Sites 2−4 were characterized by slightly alkaline waters (pH 7.2−7.5) with low-medium hardness (90−98 mg CaCO_3_/L), alkalinity (86−92 mg CaCO_3_/L) and conductivity (222−241 μS/cm). In contrast, site 1 was characterized by slightly acidic waters (pH 5.5) with very low hardness (4.3 mg CaCO_3_/L), alkalinity (3.4 mg CaCO_3_/L) and conductivity (19 μS/cm), due to major inflow from Florence Creek (Figure 1). The dichotomy in ionic composition between sites 1 and 2−4 stems largely from differences in lithology, where site 1 drains sandstone, while sites 2−4 drain carbonaceous slates. The levels of suspended particulate matter in surface waters, from all sites, were very low (1.0−1.2 mg/L), indicating that ion concentrations were largely in the dissolved and/or colloidal phase. Dissolved organic matter, a key indicator of the metal complexing capacity of the water, was relatively low (2.3−3.5 mg/L as DOC) and comparable among sites. The mean concentrations of key metal contaminants (Co, Cu, Mn, Ni, Pb, U and Zn) were highest at site 3, ranging from a factor of two for Pb, to a factor of 60 for Zn (Table 1), relative to those measured from the uncontaminated sites (pooled data for sites 1 and 4 ). The concentrations of key metal contaminants at site 2 were 2−7 times lower than site 3, but 2−10 times higher than the pooled uncontaminated sites (Table 1). Hardness-corrected metal guideline values for water quality in the Finniss River [24], classified as a slightly-moderately disturbed freshwater ecosystem, were exceeded by a factor of ten for Cu, and a factor of two for Zn, at site 3 (Table 1).

The sediment chemistry at each site is provided in Table 2. The mean concentrations of key metal contaminants (Co, Cu, Mn, Ni, Pb, U and Zn) were highest at site 3, ranging from a factor of three for Mn to a factor of 32 for Cu (Table 2), relative to those measured from the uncontaminated sites (pooled data for sites 1 and 4). The concentrations of key metal contaminants at site 2 were a 2−11 times lower than site 3 (Table 2), but only 1.4−3.1 times higher than the pooled uncontaminated sites (Table 2). Sediment concentrations of Cu and Ni at site 3 exceeded their upper threshold sediment quality guideline values [24], indicating a high probability of biological effect (Table 2), while sediment concentrations of Zn at site 3 exceeded their lower threshold guideline values, indicating a low-medium probability of biological effect (Table 2). Like Ni, the concentration of Co in sediments at site 3 was similarly elevated, relative to the pooled uncontaminated sites, but there is currently no national sediment guideline value to assess its probable biological effect.

**Table 2:** Metal concentrations in sediments from the Finniss River^a^.

	Site 4 (–15 km)	Site 3 (4 km)	Site 2 (15 km)	Site 1 (30 km)	SQG (low^b^/high^c^)
Fe	22890 (2570)	31340 (3920)	24400 (2980)	20500 (1390)	—
Mn	93 (13)	276 (31)	124 (15)	87 (10)	—
Cu	9.5 (1.4)	274* (34)	25 (3.1)	7.7 (1.0)	65/270
Zn	13 (1.6)	**259** (30)	33 (3.8)	11 (1.3)	200/410
Ni	12 (1.5)	93* (13)	20 (2.6)	10 (1.5)	21/52
Co	7.0 (1.3)	79 (12)	20 (3.2)	5.8 (1.1)	—
U	4.5 (0.8)	91 (13)	9.9 (1.5)	4.0 (0.7)	104/5870
Pb	13 (2.0)	49 (8.2)	16 (2.5)	10 (1.7)	50/220
Cd	0.033 (0.01)	0.051 (0.01)	0.038 (0.01)	0.029 (0.01)	1.5/10

a Mean (and standard deviation) values (expressed as mg/kg dry weight) based on data from 1996 to 2005 (n = 4–6), including data from this study. Values represent weak acid exchangeable metals associated with the clay/silt/sand (<2 mm) fraction.

b Sediment quality guideline (SQG) value below which there is a low probability of biological effect (Cu, Zn, Ni, Pb and Cd – [24]; U – [48]). Sediment metal concentrations that exceed this value are indicated with bold text.

c Sediment quality guideline (SQG) value above which there is a high probability of biological effect (Cu, Zn, Ni, Pb and Cd – [34]; U – [48]). Sediment metal concentrations that exceed this value are indicated with an asterisk(*).

### Annual cycle of surface water contamination

The mean Cu concentration in surface water at site 3 (35 μg/L; Table 1), based on several measured samples from 1989−2005 (combined wet/dry seasons), is markedly lower than the estimated daily maxima during the wet season (e.g. Cu peaks of 1150, 890 and 435 μg/L were measured during the 1994−1995 wet season as a typical example; Figure S1)), yet substantially higher than the mean background Cu concentration from Site 4 (0.80 μg/L; Table 1). Peak Cu concentrations at site 3 over the annual cycle were 12−33 times higher than the mean Cu concentration measured during long-term sampling at the site. It is apparent that some Cu at site 3 remained as unflushed contaminant from the terminal East branch (Figure 1) flow and/or was re-dissolved from sediments or derived from base flow contributions from the East branch (which occur at rates too low to be monitored as surface flow).

### Metal speciation in surface waters

The calculated speciation of Co, Cu, Mn, Ni, U and Zn in the surface waters of the Finniss River at each sampling site, is provided in Table 3. The free metal ion (e.g. Co^2+^), generally considered to be a good predictor of the bioavailable metal fraction, was calculated to be the major proportion of Co (83−89%), Mn (85−91%), Ni (70−79%) and Zn (78−86%), but accounted for only a very small proportion of Cu (0.2−1.9%) and U (<0.1−0.4% as UO_2_). The speciation of dissolved Cu was dominated by binding with DOM (92−98%), whereas the speciation of U was dominated by binary and ternary uranyl carbonate complexes at sites 2−4 (>99%) and binding to DOM at site 1 (98%) (Table3). More importantly, the proportion of the free metal ion was generally consistent amongst all sites. When the filtered metal concentrations at each site are corrected for the proportion of the free metal ion, the concentrations are still highest at site 3.

**Table 3:** Metal speciation calculations in surface waters of the Finniss River^a^.

Metal species^b^	Site 4 (–15 km)	Site 3 (4 km)	Site 2 (15 km)	Site 1 (30 km)
***Cobalt***				
Co^2+^	86.3 (2.3)	88.9 (1.8)	83.3 (2.2)	87.6 (5.4)
CoCO_3_	4.9 (0.1)	4.1 (0.1)	8.2 (0.2)	<0.1
CoHCO_3_^+^	2.7 (0.1)	2.8 (0.1)	2.8 (0.1)	<0.1
CoSO_4_	0.3 (0.01)	0.5 (0.01)	0.3 (0.01)	<0.1
Co-DOM	5.6 (2.2)	3.6 (1.9)	5.0 (2.3)	12.3 (5.3)
***Copper***				
Cu^2+^	0.2 (0.1)	1.4 (0.4)	0.2 (0.1)	1.9 (0.6)
CuCO_3_	1.4 (1.2)	6.7 (1.8)	1.8 (1.4)	<0.1
Cu-DOM	98.3 (2.7)	91.5 (2.4)	97.9 (2.6)	98.0 (5.5)
***Manganese***				
Mn^2+^	89.2 (1.6)	90.0 (1.2)	85.3 (1.1)	91.0 (2.8)
MnCO_3_	5.3 (0.1)	4.4 (0.1)	8.9 (0.1)	<0.1
MnHCO_3_^+^	3.2 (0.1)	3.3 (0.1)	3.4 (0.1)	<0.1
MnSO_4_	0.2 (0.01)	0.4 (0.01)	0.3 (0.01)	<0.1
Mn-DOM	1.9 (0.6)	1.8 (0.7)	1.9 (0.8)	8.9 (3.5)
***Nickel***				
Ni^2+^	70.4 (6.1)	79.3 (5.3)	70.2 (4.5)	77.2 (12.1)
NiCO_3_	6.8 (0.6)	6.4 (0.4)	11.7 (0.7)	<0.1
NiHCO_3_^+^	2.3 (0.2)	2.7 (0.2)	2.5 (0.1)	<0.1
NiOH^+^	0.3 (0.03)	0.3 (0.02)	0.5 (0.05)	<0.1
NiSO_4_	0.2 (0.02)	0.4 (0.03)	0.3 (0.03)	<0.1
Ni-DOM	19.9 (6.8)	8.4 (6.1)	14.7 (5.4)	22.7 (8.1)
***Uranium***				
UO_2_^2+^	<0.1	<0.1	<0.1	0.4 (0.1)
UO_2_OH^+^	<0.1	<0.1	<0.1	0.8 (0.1)
UO_2_CO_3_	0.7 (0.01)	0.9 (0.1)	0.2 (0.01)	0.4 (0.1)
UO_2_(CO_3_)_2_^2−^	6.8 (0.1)	8.2 (0.1)	3.7 (0.1)	<0.1
UO_2_(CO_3_)_3_^4−^	1.4 (0.1)	1.5 (0.1)	1.4 (0.1)	<0.1
UO_2_Mg(CO_3_)_3_^2−^	8.9 (0.1)	9.3 (0.1)	8.9 (0.1)	<0.1
UO_2_Ca(CO_3_)_3_^2−^	43.5 (0.3)	42.8 (0.3)	44.3 (0.3)	<0.1
UO_2_Ca_2_(CO_3_)_3_	38.3 (0.4)	37.0 (0.4)	41.3 (0.4)	<0.1
UO_2_-DOM	0.3 (0.1)	0.2 (0.05)	0.1 (0.03)	98.1 (4.1)
***Zinc***				
Zn^2+^	81.9 (5.0)	85.6 (3.1)	78.0 (4.8)	86.2 (4.1)
ZnCO_3_	4.9 (0.1)	4.1 (0.1)	8.0 (0.5)	<0.1
ZnHCO_3_	3.6 (0.2)	3.7 (0.1)	3.7 (0.2)	<0.1
ZnOH^+^	1.5 (0.1)	1.3 (0.1)	2.2 (0.1)	<0.1
ZnSO_4_	0.2 (0.01)	0.4 (0.03)	0.3 (0.03)	<0.1
Zn-DOM	7.7 (2.3)	4.8 (1.4)	7.6 (2.5)	13.7 (5.1)

a Mean (and standard deviation) values.

b Each metal species is shown as a percentage of its measured concentration (0.4 μm filtered). Metal species comprising <0.3% of total metal were excluded for clarity. DOM: dissolved organic matter.

### Metal concentrations in fish tissues (bone, liver and muscle)

Despite marked differences in the surface water chemistry of sites 1 and 4 (both uncontaminated sites), the tissue metal concentrations of both fish species were not significantly different (*P*≤0.05), thus data from both sites were pooled for univariate statistical comparisons. The mean concentrations of Ca, Co, Cu, Mg, Mn, Ni, Pb, U and Zn in bone, liver and muscle from bony bream and black catfish at the more contaminated site 3, were significantly (*P*≤0.05) lower than those from the uncontaminated sites (1 and 4) and the less contaminated site 2 (Table 4). Similarly, the mean concentrations of Na in liver and muscle (but not bone) from both fish species was lowest at site 3, relative to the other sites (Table 4). The mean concentrations of Co, Cu, Mn, Ni, Pb, U and Zn in the bone, liver and muscle of both fish species from site 3 were 17−50%, 18−49% and 15−52% lower, respectively, than those from the uncontaminated sites (pooled data for sites 1 and 4) (Table 4). The mean tissue concentrations of Cu and Zn showed the greatest decreases (44−52%), while mean tissue concentrations of Mn and Pb showed the smallest decreases (15−21%). The mean concentrations of Ca, Mg and Na in the bone, liver and muscle of both fish species from site 3 were 8−12%, 13−14% and 8−12% lower, respectively, than those from the uncontaminated sites (pooled data for sites 1 and 4) (Table 4). There were no significant (*P*>0.05) differences between bony bream and black catfish in their bone or liver metal concentrations; however, metal concentrations in the muscle of black catfish were typically 2−3-fold lower than in bony bream. For the less contaminated site 2, where Cu and Zn concentrations were elevated in surface water and/or sediment by factors of 3−10, relative to the uncontaminated sites (1 and 4), there were no significant (*P*>0.05) increases in the Cu or Zn tissue levels for either fish species.

**Table 4:** Metal concentrations in bone, liver and muscle (flesh) of bony bream and black catfish.

	Bony bream^a^			Black catfish^a^		
	Controls^b^	Site 2	Site 3	Controls^b^	Site 2	Site 3
***Bone***						
Co	0.54 (0.05)	0.52 (0.05)	**0.40** c (0.06)	0.58 (0.05)	0.56 (0.05)	**0.44** (0.04)
Cu	1.46 (0.09)	1.41 (0.09)	**1.02** (0.11)	1.53 (0.13)	1.44 (0.12)	**1.06** (0.10)
Mn	92.7 (7.2)	87.9 (8.3)	**78.0** (7.4)	97.2 (8.7)	93.8 (7.6)	**80.8** (6.2)
Ni	1.57 (0.09)	1.50 (0.10)	**1.18** (0.10)	1.53 (0.12)	1.46 (0.12)	**1.14** (0.09)
Pb	0.73 (0.06)	0.71 (0.08)	**0.63** (0.07)	0.70 (0.05)	0.68 (0.05)	**0.60** (0.04)
U	1.11 (0.08)	1.05 (0.10)	**0.86** (0.07)	1.13 (0.08)	1.08 (0.09)	**0.89** (0.07)
Zn	43.7 (3.1)	41.2 (5.0)	**28.6** (3.5)	46.1 (4.0)	44.6 (4.1)	**30.7** (3.0)
Ca^d^	246 (8.2)	241 (8.8)	**226** (9.9)	251 (8.1)	247 (7.4)	**232** (6.7)
Mg^d^	1.81 (0.08)	1.77 (0.10)	**1.65** (0.10)	1.75 (0.09)	1.70 (0.10)	**1.57** (0.09)
Na^d^	2.61 (0.10)	2.56 (0.11)	2.48 (0.11)	2.70 (0.10)	2.64 (0.10)	2.55 (0.10)
***Liver***						
Co	6.28 (0.25)	6.00 (0.24)	**4.73** (0.27)	6.39 (0.25)	6.10 (0.24)	**4.81** (0.23)
Cu	35.1 (4.7)	33.1 (5.4)	**23.9** (4.8)	32.9 (4.1)	31.0 (3.7)	**22.4** (4.0)
Mn	16.0 (1.6)	15.1 (1.9)	**13.0** (1.7)	14.4 (1.3)	13.7 (1.4)	**11.9** (1.1)
Ni	6.66 (0.65)	6.30 (0.86)	**4.78** (0.58)	6.37 (0.55)	6.04 (0.72)	**4.67** (0.59)
Pb	1.79 (0.11)	1.67 (0.12)	**1.50** (0.15)	1.90 (0.13)	1.83 (0.15)	**1.61** (0.14)
U	2.31 (0.14)	2.18 (0.14)	**1.80** (0.14)	2.13 (0.15)	2.02 (0.13)	**1.64** (0.14)
Zn	114 (11)	106 (10)	**77.9** (9.1)	126 (13)	119 (10)	**84.4** (8.4)
Ca^d^	0.97 (0.06)	0.95 (0.07)	**0.85** (0.07)	1.05 (0.06)	1.02 (0.07)	**0.93** (0.06)
Mg^d^	0.89 (0.07)	0.85 (0.05)	**0.77** (0.06)	0.97 (0.07)	0.93 (0.07)	**0.85** (0.06)
Na^d^	7.73 (0.54)	7.60 (0.56)	**6.72** (0.58)	7.39 (0.51)	7.25 (0.52)	**6.54** (0.52)
***Muscle***						
Co	0.24 (0.02)	0.22 (0.04)	**0.18** (0.02)	0.128 (0.011)	0.119 (0.012)	**0.094** (0.009)
Cu	1.64 (0.11)	1.55 (0.14)	**1.09** (0.12)	0.74 (0.06)	0.71 (0.06)	**0.50** (0.05)
Mn	16.8 (1.4)	15.9 (1.5)	**14.0** (1.3)	8.63 (0.54)	8.31 (0.72)	**7.31** (0.67)
Ni	0.55 (0.04)	0.51 (0.05)	**0.41** (0.04)	0.23 (0.02)	0.22 (0.02)	**0.17** (0.02)
Pb	0.118 (0.009)	0.112 (0.012)	**0.099** (0.010)	0.045 (0.004)	0.044 (0.004)	**0.039** (0.004)
U	0.040 (0.004)	0.038 (0.006)	**0.031** (0.004)	0.013 (0.001)	0.012 (0.001)	**0.010** (0.001)
Zn	22.0 (1.8)	20.4 (1.7)	**15.2** (1.7)	10.50 (1.0)	9.62 (0.92)	**6.90** (0.75)
Ca^d^	98.1 (3.8)	95.6 (4.3)	**86.0** (4.1)	56.7 (1.9)	54.9 (1.9)	**50.6** (2.0)
Mg^d^	150 (3.7)	154 (4.6)	**139** (4.0)	78.6 (2.2)	76.8 (2.2)	**72.6** (2.3)
Na^d^	39.1 (2.5)	39.8 (3.0)	**33.6** (2.6)	23.8 (1.4)	23.2 (1.4)	**21.3** (1.6)

a Mean (and standard deviation) values (mg/kg dry weight) for bone, liver and muscle.

b Data for uncontaminated (control) sites 1 and 4 were pooled (n = 11 for bony bream, and n = 8 for black catfish).

c Values in bold (contaminated site 3) were significantly (P≤0.05) lower than for pooled uncontaminated (control) sites. For sites 2 or 3, n = 5 for bony bream and black catfish.

d Mean (and standard deviation) values (g/kg dry weight) for bone, liver and muscle.

**Figure 2:**
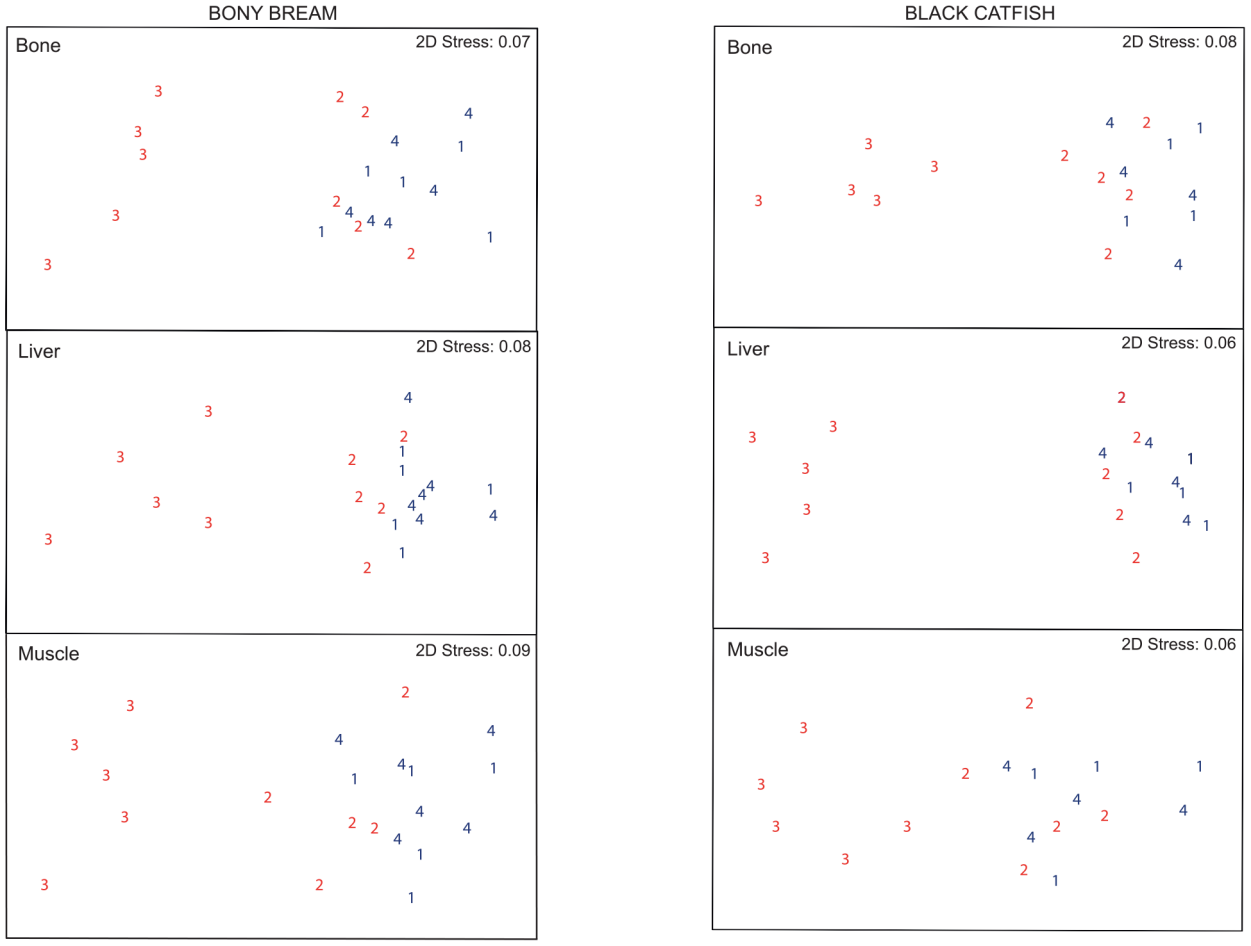
Ordination (nMDS) plots of metals in fish tissues . Ordination (2D) plots of metals in fish tissues (bone, liver and muscle) at each sampling site for (a) bony bream and (b) black catfish. Sites 1 and 4 (uncontaminated (control) sites) are shown in navy blue, whilst site 3 (the most contaminated site) and site 2 (less contaminated than site 3) is shown in red. Site 3, the most contaminated, is clearly separated from the other sites. Centroids of singles sites are represented.

Ordination (nMDS) plots for each tissue and fish species are given in Figure 2, with stress values (0.06−0.09) indicating good ordinations, with no real prospect of misinterpretation [23]. In each of the six ordinations, samples from the more contaminated site 3 were distant from all other samples. Samples from the less contaminated site 2 were in greater proximity to samples from the uncontaminated sites (1 and 4), but were also typically closer to site 3 samples than those from uncontaminated sites, particularly for bony bream and black catfish liver. The plots are consistent with a gradation of pollution effect, whereby metal levels in fish tissues decrease as the degree of metal exposure increases. The significant (*P*≤0.003) global R values (0.42−0.52) from ANOSIM confirmed that (i) in all six analyses, the site 3 samples were significantly (*P*≤0.002) different from both the uncontaminated sites (1 and 4) and the less contaminated site 2, (ii) site 2 samples were not significantly (*P*>0.05) different from either of the two uncontaminated sites, with the exception of bream liver which was significantly (*P* = 0.048) different from site 4, and (iii) samples from the two uncontaminated sites were not significantly (*P*>0.05) different.

### Relationships between metal concentrations in fish tissues and metal concentrations in surface waters and sediments

Highly significant (*P*≤0.05) inverse linear relationships were found between metal concentrations in bone, liver and muscle and metal concentrations in surface water and sediments, for both fish species (Table 5). The lowest tissue metal concentrations were evident for bony bream and black catfish at site 3, which also had the highest metal concentrations in surface waters and sediments. Metal concentrations in surface water and sediment can largely, and equally, explain the variability in metal concentrations in bone, liver and muscle, based on the high *r* ^2^ values (0.850−0.999) of the linear regressions (Table 5). For example, the Cu concentrations in surface waters and sediments explained 99% and 98%, respectively, of the variability in Cu liver concentration for bony bream, and 97% and 96%, respectively, of the variability in Cu liver concentration for black catfish.

**Table 5:** Linear regressions (*P*≤0.05; n = 4) where metal concentrations in surface water or sediments predict metal concentrations in bone, liver and muscle (flesh) of bony bream and black catfish.

	Bony bream				Black catfish			
	Surface water (SW)		Sediment (SED)		Surface water (SW)		Sediment (SED)	
	Regression equation	*r* ^2^	Regression equation	*r* ^2^	Regression equation	*r* ^2^	Regression equation	*r* ^2^
***Bone***								
Co	–0.00934(SW) + 0.549	0.987	–0.00202(SED) + 0.560	0.992	–0.00917(SW) + 0.586	0.992	–0.00197(SED) +0.596	0.988
Cu	–0.0127(SW) + 1.47	0.996	–0.00161(SED) + 1.46	0.993	–0.0134(SW) + 1.53	0.990	–0.00170(SED) + 1.53	0.971
Mn	–0.214(SW) + 98.7	0.980	–0.147(SED) + 105	0.987	–0.129(SW) + 101	0.964	–0.0892(SED) + 105	0.983
Ni	–0.0236(SW) + 1.58	0.989	–0.00467(SED) + 1.61	0.977	–0.0233(SW) + 1.53	0.991	–0.00463(SED) + 1.57	0.987
Pb	–1.22(SW) + 0.831	0.852	–0.00212(SED) + 0.753	0.850	–1.469(SW) + 0.821	0.982	–0.00265(SED) + 0.729	0.947
U	–0.319(SW) + 1.15	0.971	–0.0350(SED) + 1.11	0.968	–0.252(SW) + 1.17	0.989	–0.00271(SED) + 1.13	0.937
Zn	–0.366(SW) + 43.9	0.982	–0.0598(SED) + 44.0	0.971	–0.376(SW) + 46.6	0.985	–0.0622(SED) + 46.8	0.990
***Liver***								
Co	–0.101(SW) + 6.31	0.994	–0.0217(SED) + 6.42	0.993	–0.101(SW) + 6.42	0.995	–0.0218(SED) + 6.53	0.999
Cu	–0.322(SW) + 35.1	0.992	–0.0406(SED) + 35.0	0.976	–0.303(SW) + 32.9	0.972	–0.0384(SED) + 32.9	0.957
Mn	–0.0254(SW) + 16.9	0.981	–0.0171(SED) + 17.6	0.953	–0.0194(SW) + 14.9	0.926	–0.0133(SED) + 15.5	0.931
Ni	–0.113(SW) + 6.69	0.988	–0.0223(SED) + 6.84	0.973	–0.102(SW) + 6.40	0.988	–0.0202(SED) + 6.54	0.982
Pb	–4.18(SW) + 2.12	0.903	–0.00726(SED) + 1.85	0.851	–4.24(SW) + 2.25	0.943	–0.00762(SED) + 1.98	0.933
U	–0.479(SW) + 2.33	0.985	–0.00514(SED) + 2.27	0.972	–0.501(SW) + 2.19	0.973	–0.00539(SED) + 2.13	0.935
Zn	–0.865(SW) + 114	0.981	–0.142(SED) + 114	0.961	–1.00(SW) + 127	0.971	–0.167(SED) + 127	0.961
***Muscle***								
Co	–0.00397(SW) + 0.241	0.951	–0.000861(SED) + 0.246	0.972	–0.00215(SW) + 0.128	0.929	–0.000464(SED) + 0.130	0.940
Cu	–0.0157(SW) + 1.64	0.997	–0.00198(SED) + 1.63	0.986	–0.00703(SW) + 0.745	0.984	–0.000814(SED) + 0.743	0.980
Mn	–0.0219(SW) + 17.4	0.941	–0.0149(SED) + 18.0	0.928	–0.0103(SW) + 8.93	0.987	–0.00706(SED) + 9.24	0.989
Ni	–0.00824(SW) + 0.548	0.966	–0.00162(SED) + 0.558	0.939	–0.00402(SW) + 0.239	0.965	–0.000797(SED) + 0.245	0.959
Pb	–0.280(SW) + 0.141	0.912	–0.000493(SED) + 0.123	0.857	–0.0867(SW) + 0.0521	0.989	–0.000158(SED) + 0.0467	0.988
U	–0.00952(SW) + 0.0415	0.982	–0.000110(SED) + 0.0401	0.928	–.00286(SW) + 0.0130	0.948	–0.0000303(SED) + 0.0126	0.872
Zn	–0.161(SW) + 21.9	0.979	–0.0264(SED) + 22.0	0.959	–0.0816(SW) + 10.3	0.976	–0.0134(SED) + 10.3	0.961

**Figure 3:**
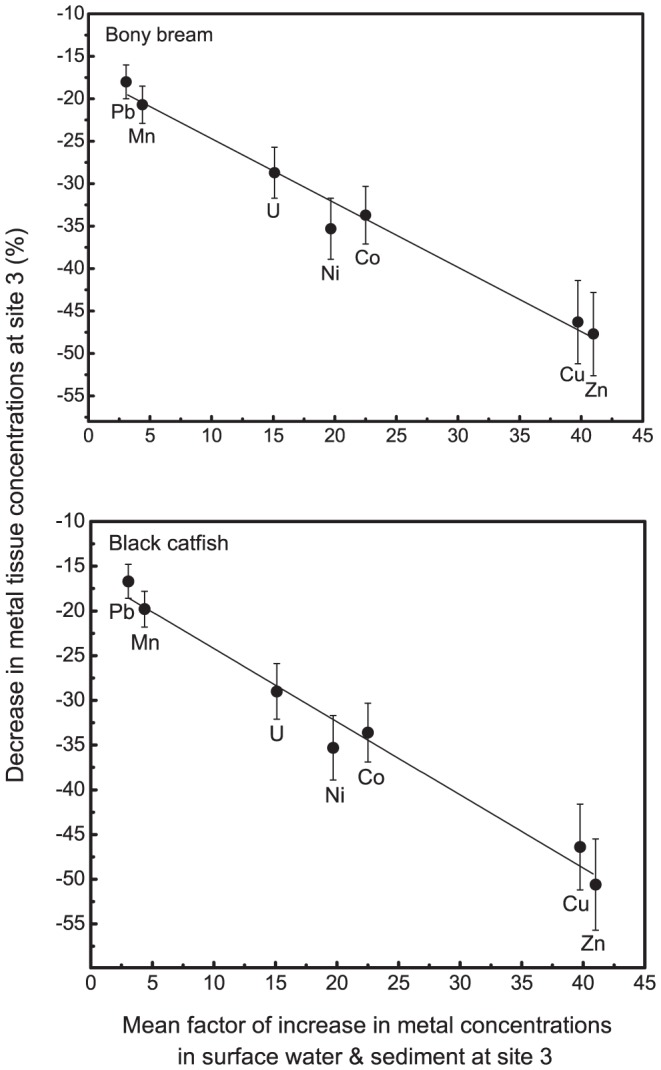
Linear regressions of metals in fish tissues versus metals in sediment and surface water. Linear regressions of the percentage decrease in metal tissue concentrations (mean of bone, liver and muscle) at site 3 (relative to control sites 1 and 4) plotted against the mean factor of increase in metal concentrations for surface water and sediment at site 3 (relative to control sites 1 and 4) for (a) bony bream (*r* ^2^ = 0.978; *P*<0.01; y = –0.749x – 17.3) and (b) black catfish (*r* ^2^ = 0.974; *P*<0.01; y = –0.819x –16.0).

The degrees of reduction in metal tissue concentrations varied amongst metals, as observed in the slopes of the linear regressions of metals levels in surface water and sediment against metal tissue concentrations, for both species (Table 5) – which are mainly determined by the site 3 values. To determine if there was any systematic relationship between the bioaccumulation of metals by fishes relative to their degree of exposure, the percentage decrease in metal tissue concentrations (pooled data for bone, liver and muscle) at site 3 (relative to pooled data from control sites 1 and 4) was regressed against the mean factor of increase in metal concentrations for surface water and sediment at site 3 (relative to pooled data from control sites 1 and 4) for bony bream and black catfish. These highly significant (*P*≤0.001) inverse linear regressions (Figure 3) indicate that the degrees of reduction in metal bioaccumulation are greater with increasing metal exposure, relative to controls. Both fish species also show the same placements of metals along the regression lines, with Cu and Zn showing the greatest reductions in bioaccumulation for the greatest increases in their environmental concentrations, and Pb and Mn the least.

## Discussion

### Reduced metal bioaccumulation with increasing environmental metal exposure

Metal concentrations in fish tissues typically increase with exposure to increasing environmental metal concentrations, although this relationship generally has a proportionality of <1 [25], [26]. However, this study shows that sampled populations of bony bream and black catfish exposed to the highest concentrations of mine-related metals (Co, Cu, Mn, Ni, Pb, U and Zn) in surface water and sediment (site 3) have, paradoxically, the lowest concentrations of these metals in their liver, bone and muscle. Fishes from the less contaminated site 2 show no significant (*P*>0.05) increases in tissue metal concentrations above background (sites 1 and 4), although environmental metal concentrations are enhanced by factors of 1.4−10; moreover their multi-metal patterns shown a greater similarity to those from site 3, compared with the uncontaminated sites (1 and 4; Figures 2a and 2b).

The concentrations of Co, Cu, Mn, Ni, Pb, U and Zn in the liver, bone and/or muscle of bony bream and black catfish from sites 1 and 4 (uncontaminated sites) were consistent with those of another study in a nearby catchment unaffected by ARD (Table S2), as well as other local and overseas freshwater fish from uncontaminated sites [27], [28], [29], [30], [31], [32], [33].

Thus, fish populations from the chronically contaminated regions have not generally responded to the enhanced environmental metal levels (surface water or sediment) by elevating their tissue metal concentrations (at least for liver, bone and muscle) as would be expected assuming constant or proportional concentration factors.

### Hypotheses to explain diminished bioaccumulation of metals with enhanced environmental metal concentrations

#### Reduced bioavailability or exposure to metals

To determine whether reduced metal concentrations in the tissues of bony bream or black catfish may be explained by reduced metal bioavailability, geochemical speciation modeling was used to estimate the potential bioavailable metal fractions in the surface waters at each site (Table 3). The results of the speciation modeling, however, show no indication of reduced bioavailability of metals at site 3, where metal concentrations are highest, relative to other sites. Hence, the lowest metal tissue concentrations in both fish species at site 3 cannot be explained, even in part, by reduced metal bioavailability in the surface waters.

Both fishes are known to ingest sediment as part of their diet. Bony bream feeds primarily on detritus and benthic algae [34], [35], [36], whereas black catfish feeds primarily on benthic invertebrates [34]. The sediment concentrations of all metals were highest at site 3 (Table 2), where tissue metal concentrations were lowest in both fish species. Therefore, reduced metal bioaccumulation under enhanced metal exposure cannot be explained by reduced bioavailability of aqueous metals or reduced sediment metal concentrations.

A homeostatic interaction between the gills and gut in the uptake of Cu, Zn and Ni in rainbow trout has been reported [37], [38], [39]. [37] found that chronic exposure to elevated levels of Cu via the diet (gut) can cause a reduction in waterborne Cu uptake (gills) in rainbow trout. In addition, [40] found evidence at the molecular level demonstrating that sea bass exposed to dietary Cu have a reduced messenger ribonucleic acid (mRNA) level of Cu-ATPase (ATP7a), a key regulatory protein of Cu homeostasis, not only in the gut, but also in the gill and liver. Based on a preliminary study of metal concentrations in selected fish dietary items (e.g. crustaceans) among the four sampling sites in the present study, [7] reported that metal concentrations in prey items were highest at site 3 (where metal tissue concentrations in fish were lowest) and lowest at sites 1 and 4. Furthermore, [39] found that pre-exposure of rainbow trout to elevated concentrations of waterborne Ni down-regulated gastrointestinal Ni uptake. The demonstrated interaction between gill and gut metal uptake requires further laboratory investigation in bony bream and black catfish, in light of offering some further explanation of the observed paradoxical results of this study.

Enhanced levels of essential macro-nutrients, such as Ca, Mg and/or Na, could reduce the uptake of trace metals from water in both fish species by the mechanism of competitive inhibition, e.g. Na inhibits Cu uptake, and Ca/Mg inhibits Cd, Co, Mn, Ni, Pb, Zn or U uptake [25], [26]. However, the surface water chemistry of the sampling sites (Table 1) indicates minimal differences in the concentrations of Ca, Mg or Na among sites 2−4; site 1 has a unique water chemistry and was excluded from comparison. Thus, differences in macro-nutrient water concentrations cannot explain the reduced trace metal concentrations in fish tissues.

Due to the motility and olfactory capacities of fish, another possible interpretation is that fishes sampled from the contaminated site 3 have recently migrated from uncontaminated regions (e.g. site 1) of the river, or have reduced residence times at the more contaminated site 3 due to avoidance behavior. Consequently, their tissues may not have equilibrated with the metal concentrations in the contaminated site(s), prior to sampling. These related scenarios are discounted for the following reasons:

- With regard to fish migration, (i) fish were sampled from the contaminated region of the river during the late dry season when pools were isolated from each other for at least four months based on recorded river heights. Hence, there was no possibility during this period for very recent migration from uncontaminated sites. Similarly, during a pre-remedial study in 1973 where fish were sampled at three periods over the dry season, when metal input to the Finniss River had ceased, and there were increased abundances of potential food resources [4], there was no recovery of either fish species, or fish community structure, resulting from migration from other regions [6]; (ii) in such a linear riverine system with no major tributaries, except Florence Creek (upstream of site 4), the most likely sources of immigrants to potentially supply fish to the contaminated sites are those in closest proximity, which are the uncontaminated (or control) sites, where both fish species have higher metal concentrations (Table 4; Figure 2). Therefore, fish tissues from the metal contaminated sites would show similar (elevated) metal levels to the control sites, rather than the significantly (*P*≤0.05) reduced tissue metal levels observed (Table 4).
- With regard to possible disequilibrium of metal concentrations in fish tissues, (i) previous Cu-64/67 radiotracer studies with both control and Cu-exposed populations of black-banded rainbowfish (*Melanotaenia nigrans*; albeit a smaller species), showed that its tissue levels equilibrated with the Cu-64/67 water concentrations within days of exposure [41], suggesting that populations of bony bream and black catfish are more likely than not to have tissue concentrations of metals approaching equilibrium with environmental concentrations, and (ii) most bony bream or black catfish from the contaminated region of the Finniss River would have to spend appreciable periods of time exposed to lower concentrations of metals in water and/or sediment than those populations from both uncontaminated sites (1 and 4). Moreover, exposure conditions would have to be different for fishes of both species collected at sites 2 and 3, because of their different surface water and/or sediment metal concentrations. It may also be expected that such notional mixed exposure regimes for fishes collected in the contaminated region of the Finniss River would result in greater variation among individuals in their tissue metal concentrations compared to control samples; however, this was not the case.
- With regard to avoidance of metal contaminants, it is relevant that at the same time of sampling in the Finniss River, five other species of local fish had actively recolonised the metal pollution gradient of its East Branch (Figure 1) to varying degrees, where surface water concentrations of Cu, Zn and Mn were up to an order of magnitude higher than in the contaminated region (site 3) of the main channel of the Finniss River [5].

In summary, although the above-mentioned explanatory hypotheses seem implausible, they cannot be completely discounted because of the absence of data on the motility and residence times of fishes collected from both control and metal contaminated sites of the Finniss River.

#### Modified metal biokinetics

Alternatively, it may be hypothesised that populations of bony bream and black catfish have modified kinetics within their metal bioaccumulation physiology during their five decades of metal exposure from the Rum Jungle mine site, via adaptation or tolerance responses. Experimental studies on aquatic organisms living in metal contaminated environments have shown reduced uptake rates of metals in exposed populations relative to controls. In a study of copper acclimation in the least killifish (*Heterandria formosa*), the rate of whole body Cu accumulation was reduced in fish acclimated to enhanced Cu water concentrations compared to controls [42]. Strains bred to be genetically resistant to Cd also showed reduced rates of Cd uptake compared to control lines, where the reduced uptake and accumulation of Cd explained about two thirds of their increase in Cd resistance [43].

More pertinent to interpreting the results of the present study are the findings of an experimental study on mechanisms of Cu tolerance of a population of black-banded rainbowfish (*Melanotaenia nigrans*). Populations of this species from the East Branch of the Finniss River had also been exposed to elevated Cu concentrations over the same period. The bioconcentration of ^64/67^Cu by *M. nigrans* from the East Branch was used to investigate the mechanism of Cu tolerance. Both long-term metal-exposed (East Branch) and reference fish populations were experimentally-exposed to 30 μg/L and 300 μg/L Cu for 24 and 48 h, respectively [41]. Copper concentrations in the head, muscle and whole body were reduced by up to a factor of two in (tolerant) fish from the East Branch, compared with reference fish, when exposed to both Cu concentrations, even after their acclimation to Cu water concentrations lower than background for two months. The mechanism of Cu tolerance was concluded to be reduced Cu uptake in the gills, rather than increased binding or elimination [41]. These factors of reduction in Cu accumulation in tolerant *M. nigrans* are similar in magnitude to the decreases in Cu tissue concentrations of bony bream and black catfish from the more contaminated site 3 (Table 4), although the mean Cu concentrations in water and sediment from site 3 are ∼40 times higher than in uncontaminated sites (1 and 4).

The reduced uptake of Cu in metal-exposed populations of *H. formosa* and *M. nigrans* adaptively reduces exposure of their internal tissues to Cu and other metals. Similarly, for both bony bream and black catfish, the inverse relationship between metal bioaccumulation and increasing environmental exposure (Figure 3) is consistent with an adaptive response to reduce metal exposure of internal tissues. This adaptive response also appears quite discerning among different metals, and in accordance with their magnitude of increase above natural levels (Figure 3).

With regard to the possible physiological mechanisms involved in the reduced rates of trace metal accumulation in bony bream and black catfish from the contaminated region of the Finniss River, the following findings are relevant. [43] previously proposed that resistance to Cd in least killifish, resulting from reduced rates of uptake, may be a consequence of changes to Ca uptake pathways, through which Cd may be absorbed, along with other divalent trace metals, such as Co, Mn, Ni, Pb U and Zn [25], [26].

The reductions in tissue concentrations of Ca, Mg and Na in bony bream and black catfish in the contaminated region (site 3), relative to controls, where there are no changes in their water or sediment concentrations amongst sites 2−4 (Table 1), is consistent with modifications to their Ca and Na uptake pathways, possibly where a reduction in their accumulation facilitates concomitant reductions in accumulation of those trace metals that are absorbed via the gills and/or gut as metabolic analogues. Many of the trace metals accumulated in bony bream and black catfish follow analogous metabolic pathways to essential macro-nutrients, particularly Ca and Na (i.e. Ca competing with Co, Mn, Ni, Pb, U and Zn, and Na competing with Cu; [25], [26]). Comparisons of Ca and Na tissue concentrations between the pooled uncontaminated sites (1 and 4) with the more contaminated site 3, showed that black catfish and bony bream had significantly (*P*≤0.05) lower mean tissue concentrations of Ca (8−12%) and Na (8−12%) in the more contaminated site (Table 4). These percentage reductions are typically lower in magnitude than those for trace metals, possibly due to stronger homeostatic regulation, but still consistent with a general inhibitory mechanism operating in the metal-tolerant populations of bony bream and black catfish.

The exposure of fish populations to metals in the Finniss River over five decades is well documented, as described above. Fish-kills were repeatedly observed prior to mine site remediation [6]. Moreover, the temporal pattern of Cu concentrations in surface waters from the most contaminated site 3 during the post-remedial period, confirms the continued exposure of fish populations to short-term spikes in Cu concentrations that are repeatedly more than two orders of magnitude above the Cu guideline value for short-term exposures (Table 1; Figure S1). Previous electrophoretic studies on allozyme frequencies and heterozygosity in tolerant *M. nigrans* exposed over the same period indicated that selection had also occurred [41]. Hence, it is plausible that the abilities of bony bream and black catfish to reduce trace metal levels in their tissues with long-term environmental metal exposure may have a genetic component, and such a determination for these species would be valuable, in light of the findings of this work.

Selection for metal tolerance in aquatic organisms may be due to either acclimation, adaptation or both [44]. Selection would favor individuals with a capacity to reduce their exposure to trace metals, as observed in the three studied fish species (i.e. bony bream and black catfish (this study) and black-banded rainbowfish [41]), possibly through reduced metal uptake, as experimentally demonstrated for Cu in black-banded rainbowfish [41] and a crustacean (*Gammarus marinus*) [45]. Although physiological acclimation cannot be excluded, evolutionary selection can result in a genetically-based tolerance, that for fish may occur within 15 or less generations [46] – well within the 50 year time-frame of metal exposure in the Finniss river. Autecology studies of fish resident in rivers in the vicinity of the Finniss River [47] indicate that both black catfish and bony bream can achieve sexual maturity within a year and are likely to spawn in sites similar to those sampled in this study. Moreover, where selection pressure for the evolution of resistance is strong, it can override low levels of gene flow in fish populations from the uncontaminated, or less contaminated, sites [44]. Adaptations to elevated metal exposure may continue to persist after such causal factors have ameliorated or disappeared [44] and surface water metal concentrations in the metal-impacted region of the Finniss river were certainly reduced after mine site remediation [6]. However, contemporary surface water and sediment metal concentrations (Tables 1 and 2) at site 3, still exceed national guideline values (by factors of up to 2–3) for protecting freshwater ecosystems [24].

## Supporting Information

Figure S1 Estimated daily Cu concentrations at site 3 over the 1994*−*1995 wet season. An estimate of a typical temporal pattern of annual contaminant exposure to fish at site 3 (Figure 1) was calculated using measured daily Cu concentrations and flow rates at two gauging stations, GS8150097 (located on the East branch of the Finniss River, 5.6 km downstream of the Rum Jungle mine site) and GS8150204 (located on the Finniss river downstream of the confluence with the East Branch, within a few hundred meters of site 3). GS8150097 is believed to capture all contaminant sources from the Rum Jungle mine. The daily Cu concentrations (mg/L) at GS8150097 were converted to daily loads (kg/day) using the mean daily flow rate (m^3^/sec x sec/day). A delay of one day was assumed as a transit time between the two gauging stations. Hence, the Cu load carried by the East Branch was divided by the flow rate measured in the Finniss River at GS8150204 a day later to estimate a Cu concentration at site 3. The background Cu concentration (0.80 μg/L, Table 1) from Site 4 was added, to derive an estimate of the daily Cu concentrations at Site 3.
(TIF)

Table S1 Total length (TL, snout to caudal fin, mm) and age (years) of bony bream and black catfish at each sampling site^a^.
(DOCX)

Table S2 Metal concentrations in liver and muscle (flesh) of bony bream and black catfish from uncontaminated (control) sites on the Finniss River and Magela Creek (forms part of a nearby uncontaminated catchment).
(DOCX)
