# Supplementary figures and images for: Diminished Metal Accumulation in Riverine Fishes Exposed to Acid Mine Drainage over Five Decades
Source: PLoS One. 2014 Mar 24;9(3):e91371. doi: 10.1371/journal.pone.0091371 (PMC3963865; doi:10.1371/journal.pone.0091371)

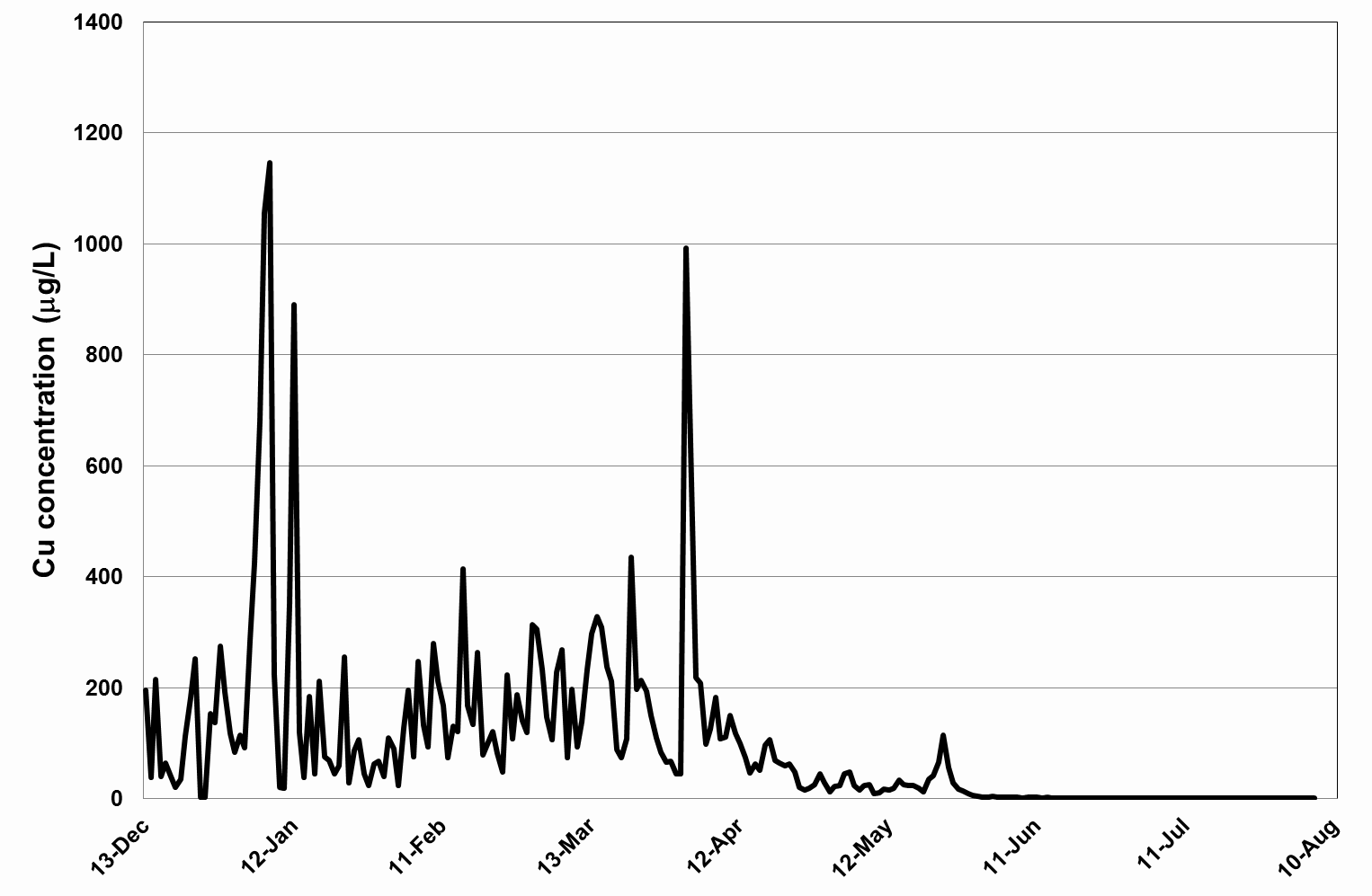

Supplement: Figure S1 — Estimated daily Cu concentrations at site 3 over the 1994−1995 wet season. An estimate of a typical temporal pattern of annual contaminant exposure to fish at site 3 (Figure 1) was calculated using measured daily Cu concentrations and flow rates at two gauging stations, GS8150097 (located on the East branch of the Finniss River, 5.6 km downstream of the Rum Jungle mine site) and GS8150204 (located on the Finniss river downstream of the confluence with the East Branch, within a few hundred meters of site 3). GS8150097 is believed to capture all contaminant sources from the Rum Jungle mine. The daily Cu concentrations (mg/L) at GS8150097 were converted to daily loads (kg/day) using the mean daily flow rate (m3/sec x sec/day). A delay of one day was assumed as a transit time between the two gauging stations. Hence, the Cu load carried by the East Branch was divided by the flow rate measured in the Finniss River at GS8150204 a day later to estimate a Cu concentration at site 3. The background Cu concentration (0.80 μg/L, Table 1) from Site 4 was added, to derive an estimate of the daily Cu concentrations at Site 3. (TIF) [file pone.0091371.s001.tif]
