# Supplementary material for: Diminished Metal Accumulation in Riverine Fishes Exposed to Acid Mine Drainage over Five Decades
Source: PLoS One. 2014 Mar 24;9(3):e91371. doi: 10.1371/journal.pone.0091371 (PMC3963865; doi:10.1371/journal.pone.0091371)
Supplement: Table S1 — Total length (TL, snout to caudal fin, mm) and age (years) of bony bream and black catfish at each sampling sitea. (DOCX) [file pone.0091371.s002.docx]

**Table S1**. Total length (TL, snout to caudal fin, mm) and age (years) of bony bream and black catfish at each sampling site^a^.

|  | **Bony bream** | | | |  | **Black catfish** | | | |
| --- | --- | --- | --- | --- | --- | --- | --- | --- | --- |
|  | Site 1 | Site 2 | Site 3 | Site 4 |  | Site 1 | Site 2 | Site 3 | Site 4 |
| Mean TL  (95% CI) | 256  (246−266) | 245  (237−253) | 254  (246−262) | 250  (244−267) |  | 316  (290−332) | 305  (295−315) | 295  (283−307) | 301  (289−313) |
| Min-max TL | 241−271 | 242−265 | 238−258 | 238−264 |  | 282−330 | 285−318 | 270−320 | 285−315 |
| Min-max age^b^ | 2.1−2.6 | 2.1−2.5 | 2.0−2.3 | 2.0−2.5 |  | 1.9−2.4 | 1.9−2.3 | 1.8−2.3 | 1.9−2.3 |
| Sample size (*n*) | 5 | 5 | 5 | 6 |  | 4 | 5 | 5 | 4 |

^a^ One-way analysis of variance revealed no significant (*P* > 0.05) differences in total length or age between sites for both fish species.

^b^ Age estimated from total length [1,2].

**References**

1. Bishop KA, Allen SA, Pollard DA, Cook MG (2001) Ecological studies on the freshwater fishes of the Alligator Rivers Region, Northern Territory. Volume III: Autecology. Research Report 4. Canberra: Supervising Scientist for the Alligator Rivers Region. Australian Government Publishing Service.
2. Puckridge JT, Walker KF (1990) Reproductive biology and larval development of a gizzard shad, *Nematolosa erebi* (Günther) (Dorosomatinae: Teleostei), in the River Murray, South Australia.
