# Supplementary material for: Diminished Metal Accumulation in Riverine Fishes Exposed to Acid Mine Drainage over Five Decades
Source: PLoS One. 2014 Mar 24;9(3):e91371. doi: 10.1371/journal.pone.0091371 (PMC3963865; doi:10.1371/journal.pone.0091371)
Supplement: Table S2 — Metal concentrations in liver and muscle (flesh) of bony bream and black catfish from uncontaminated (control) sites on the Finniss River and Magela Creek (forms part of a nearby uncontaminated catchment). (DOCX) [file pone.0091371.s003.docx]

**Table S2**. Metal concentrations in liver and muscle (flesh) of bony bream and black catfish from uncontaminated (control) sites on the Finniss River and Magela Creek (forms part of a nearby uncontaminated catchment).

|  | **Bony Bream^a^** | | | |  | **Black catfish^a^** | | | |
| --- | --- | --- | --- | --- | --- | --- | --- | --- | --- |
|  | Finniss River^b^ |  |  | Magela Creek^c^ |  | Finniss River^b^ |  |  | Magela Creek^c^ |
|  | Site 1 | Site 4 |  | Composite sites |  | Site 1 | Site 4 |  | Composite sites |
| ***Liver*** |  |  |  |  |  |  |  |  |  |
| Cu | 35.4 (4.7) | 34.8 (5.4) |  | 42.1 (12.2) |  | 32.1 (3.7) | 33.6 (4.1) |  | 33.4 (8.1) |
| Mn | 16.4 (2.3) | 16.1 (1.7) |  | 19.2 (4.7) |  | 14.7 (1.3) | 14.1 (1.3) |  | 15.8 (4.5) |
| Pb | 1.76 (0.11) | 1.82 (0.17) |  | 1.73 (0.34) |  | 1.94 (0.13) | 1.86 (0.14) |  | 2.09 (0.31) |
| U | 2.30 (0.16) | 2.22 (0.16) |  | 1.88 (0.36) |  | 2.17 (0.14) | 2.08 (0.15) |  | 1.75 (0.30) |
| Zn | 112 (12) | 116 (12) |  | 105 (17) |  | 129 (13) | 123 (11) |  | 133 (21) |
| ***Muscle*** |  |  |  |  |  |  |  |  |  |
| Co | 0.246 (0.022) | 0.238 (0.020) |  | 0.252 (0.065) |  | 0.125 (0.017) | 0.131 (0.012) |  | 0.118 (0.038) |
| Cu | 1.63 (0.12) | 1.65 (0.12) |  | 1.61 (0.22) |  | 0.753 (0.069) | 0.725 (0.062) |  | 0.837 (0.163) |
| Mn | 17.1 (1.4) | 16.6 (1.4) |  | 20.3 (5.8) |  | 8.69 (0.80) | 8.57 (0.71) |  | 8.39 (2.12) |
| Ni | 0.547 (0.048) | 0.553 (0.042) |  | 0.497 (0.093) |  | 0.243 (0.022) | 0.233 (0.021) |  | 0.312 (0.078) |
| Pb | 0.117 (0.012) | 0.121 (0.012) |  | 0.127 (0.039) |  | 0.0453 (0.0044) | 0.0447 (0.0040) |  | 0.065 (0.021) |
| U | 0.0400 (0.0056) | 0.0405 (0.0038) |  | 0.0361 (0.0086) |  | 0.0130 (0.0013) | 0.0124 (0.0014) |  | 0.0120 (0.004) |
| Zn | 21.7 (2.3) | 22.3 (1.8) |  | 26.4 (4.8) |  | 10.5 (1.0) | 10.1 (1.0) |  | 13.4 (4.1) |

^a^ Mean (and standard deviation) values (mg/kg dry weight).

^b^ Data from this study (Bony bream: n = 5 for site 1 and n = 6 for site 4; Black catfish: n = 4 for sites 1 and 4).

^c^ Data calculated from [3] and the Environmental Research Institute of the Supervising Scientist (unpublished data) (n = 2 for liver and n = 3-6 for muscle.

**Reference**

1. Ryan B, Martin P, Humphrey C, Pidgeon R, Bollhofer A, Fox T, Medley P (2005) Radionuclides and metals in fish and freshwater mussels from Mudginberri and Sandy billabongs, Alligator Rivers Region. IR 498, Canberra: Australian Governmen
